# Variation in the Chemical Composition of Small Cranberry (*Vaccinium oxycoccos* L.) Fruits Collected from a Bog-Type Habitat in Lithuania

**DOI:** 10.3390/ijms26146956

**Published:** 2025-07-20

**Authors:** Mindaugas Liaudanskas, Rima Šedbarė, Irmantas Ramanauskas, Valdimaras Janulis

**Affiliations:** 1 Department of Pharmacognosy, Faculty of Pharmacy, Lithuanian University of Health Sciences, LT-50162 Kaunas, Lithuania; rima.sedbare@lsmu.lt (R.Š.); valdimaras.janulis@lsmu.lt (V.J.); 2 Institute of Pharmaceutical Technologies, Faculty of Pharmacy, Lithuanian University of Health Sciences, LT-50162 Kaunas, Lithuania; 3 Department of Languages and Education, Faculty of Medicine, Lithuanian University of Health Sciences, LT-50275 Kaunas, Lithuania; irmantas.ramanauskas2@lsmu.lt

**Keywords:** anthocyanins, antioxidant activity in vitro, flavonols, hydroxycinnamic acid derivatives, proanthocyanidins, ripening period, triterpene compounds, *Vaccinium oxycoccos* L.

## Abstract

This study revealed variations in the composition and in vitro antioxidant activity of proanthocyanidins, hydroxycinnamic acid derivatives, flavonols, anthocyanins, and triterpene compounds in small cranberry fruit samples collected from a bog-type natural habitat in Lithuania during intensive ripening of the fruit. The highest total amounts of identified flavonols were determined at the beginning of fruit ripening on September 10 (1232.84 ± 31.73 µg/g). The highest total amounts of proanthocyanidins (1.85 ± 0.02 mg EE/g, *p* < 0.05), anthocyanins (4096.79 ± 5.93 µg/g, *p* < 0.05), and triterpene compounds (8248.46 ± 125.60 µg/g, *p* < 0.05) were detected in small cranberry fruit samples collected in the middle of the ripening period (September 16–18). The most potent in vitro antiradical and reducing activity was found in extracts of small cranberry fruit collected on September 10 (95.25 ± 1.15 µmol TE/g and 159.26 ± 0.77 µmol/g, respectively). The strongest correlation was found between the total content of hydroxycinnamic acid derivatives in the small cranberry fruit samples and the in vitro reducing activity of their extracts (0.858, *p* < 0.01). Among the individual compounds, the strongest correlation was observed between the amounts of isoquercitrin and guaijaverin in *V. oxycoccos* fruit samples and the in vitro reducing activity as assessed by the CUPRAC method (0.844, *p* < 0.01 and 0.769, *p* < 0.05, respectively).

## 1. Introduction

The use of herbal medicines and dietary supplements has increased significantly over the last decades [1]. This trend has led to the growing popularity of medicinal plant materials, which accumulate biologically active compounds with a wide range of pharmacological effects. Such compounds protect structural molecules in the human body from damage caused by various exogenous and endogenous agents. The growing interest in plant materials encourages a wider application of modern methods for studying their chemical composition and biological effects. It also promotes the development of research-based recommendations for the use of botanical preparations. Plant materials and products derived from plants of the family *Ericaceae* Juss. have strong antioxidant properties [2], making them important for practical medicine, healthy eating, cosmetics, and the food industry.

Small cranberry (*Vaccinium oxycoccos* L.), a member of the *Ericaceae* family and the genus *Vaccinium* L., is widespread in the cold and temperate zones of Eurasia and North America [3]. In Lithuania, the largest cranberry habitats are located in areas of raised bogs and intermediate wetlands. In Lithuania, land reclamation in the second half of the 20th century drained a large number of raised bogs and intermediate wetlands, which reduced the areas of the natural habitats of small cranberries [4]. Conservation of wetlands is relevant for the preservation and development of small cranberry habitats. In Lithuania, wetlands are distributed throughout the country; most of them are small and cover areas of up to 50 ha [5]. Small cranberry fruits have been used for food and folk medicine in the Baltic Sea region since ancient times and have attracted the attention of scientists as well as health and nutrition experts. In food processing, fruits are used to produce jams, juices, sauces, jellies, and soft drinks [6]. In folk medicine, the medicinal plant material of small cranberries is used to treat diseases of the digestive and urinary systems, while the juice is used to treat rheumatism, throat diseases, general body weakness, and avitaminosis [7]. Small cranberry fruits have been found to contain flavonols (quercetin and myricetin glycosides), proanthocyanidins, hydroxycinnamic acid derivatives, anthocyanins, triterpene compounds, organic acids, and vitamins [4,8]. Triterpene compounds of small cranberry fruits exhibit pronounced anti-inflammatory activity [9]. Flavonols, proanthocyanidins, phenolic acids, and anthocyanins have been found to have antioxidant effects [10]. Plant materials and preserves rich in phenolic compounds have been shown to be of value in the treatment of oncological, cardiovascular, and neurodegenerative diseases [11].

Thus, it is relevant to investigate the qualitative and quantitative variation in the composition of biologically active compounds in plant materials by applying modern techniques used for the analysis of plant secondary metabolites. Changes in the qualitative and quantitative composition of secondary metabolites during the ripening of small cranberry fruit influence their biological effects, determining the qualitative composition and the organoleptic properties of the botanical material [12]. It is also important to determine and evaluate the chemical composition of small cranberry fruits growing under Lithuanian climatic conditions in order to identify (i) the compounds that determine their antioxidant effect and (ii) the patterns of variation in the in vitro antiradical and reducing activity of the bioactive compounds in small cranberry fruit extracts. Detailed studies on the phytochemical composition of small cranberry fruit will ensure the preparation of high-quality small cranberry plant material, will allow for the rational use of plant resources by determining the optimum timing of small cranberry fruit picking and will provide research-based recommendations for the rational harvesting of small cranberry fruit.

The aim of the study was to determine the variation in the phytochemical composition and in vitro antioxidant activity of small cranberry fruit samples collected in a bog-type natural habitat in Lithuania during intensive ripening of fruit.

## 2. Results and Discussion

### 2.1. Determination of the Composition of Total Proanthocyanidins, Hydroxycinnamic Acid Derivatives, Individual Flavonols, and Chlorogenic Acid

Changes in the qualitative and quantitative composition of secondary metabolites in small cranberry fruits during their development and ripening determine their quality and may influence their effects on biological systems in the human body. It is thus relevant to investigate the variation in the qualitative and quantitative composition of phenolic compounds in small cranberry fruits growing in natural habitats in the territory of the Republic of Lithuania during their development and ripening.

Anthocyanins, flavonols, proanthocyanidins, and phenolic acids are the main groups of biologically active compounds in small cranberry plant materials [10]. Kylli et al. [13], using ultra-high-performance liquid chromatography coupled with photodiode array and fluorescence detectors (UHPLC-PDA-FL), found that proanthocyanidins accounted for 63%, anthocyanins for 16%, flavonols for 14%, hydroxycinnamic acids for 7%, and hydroxybenzoic acids for 0.05% of the total amount of phenolic compounds in *V. oxycoccos* fruit samples.

The proanthocyanidins found in small cranberry fruit are important for the prevention and complementary treatment of urinary tract infections [3-Jurikova]. A complex of type A trimeric proanthocyanidins inhibits the adhesion of a uropathogenic urinary-tract-infection-causing *Escherichia coli* strain to urinary epithelial cell receptors [14]. Herbal preparations of some small cranberry species have traditionally been used to alleviate symptoms of mild recurrent lower urinary tract infections [7]. Proanthocyanidins are found in plants of the genus *Vaccinium*, but some proanthocyanidins are analytical markers of the chemical composition of small cranberry fruit raw material [15].

In our study, we used spectrophotometric analysis to evaluate the proanthocyanidin content of small cranberry fruit samples collected during their development and ripening. The total proanthocyanidin content of the samples was expressed as the (–)-epicatechin equivalent (EE) and ranged from 1.33 mg EE/g to 1.85 mg EE/g. The mean total proanthocyanidin content of the samples analyzed was calculated to be 1.55 ± 0.17 mg EE/g. The highest total proanthocyanidin content (1.85 ± 0.02 mg EE/g, *p* < 0.05) was found in the small cranberry fruit samples collected on September 18. The conditions during the fruit ripening period that influenced the changes in proanthocyanidin content were primarily related to the intensive fruit ripening stage [16,17], which is characterized by significant physiological and biochemical changes in the fruit. During the intensive fruit ripening stage, the metabolic activity of the plant increases, leading to enhanced synthesis and accumulation of secondary metabolites, including proanthocyanidins. Environmental factors such as temperature [18], light intensity [19], and water availability [20] during ripening may also contribute to the regulation of proanthocyanidin biosynthesis by affecting enzymatic activities and gene expression related to flavonoid pathways. Therefore, the observed increase in proanthocyanidin content reflects the combined effect of the fruit’s developmental stage and the prevailing environmental conditions. The lowest total proanthocyanidin content (1.33 ± 0.04 mg EE/g) was found in the *V. oxycoccos* fruit samples collected on October 3, but it was not statistically significantly different from the total proanthocyanidin content of small cranberry fruit samples collected on September 11 or September 16.

**Figure 1:**
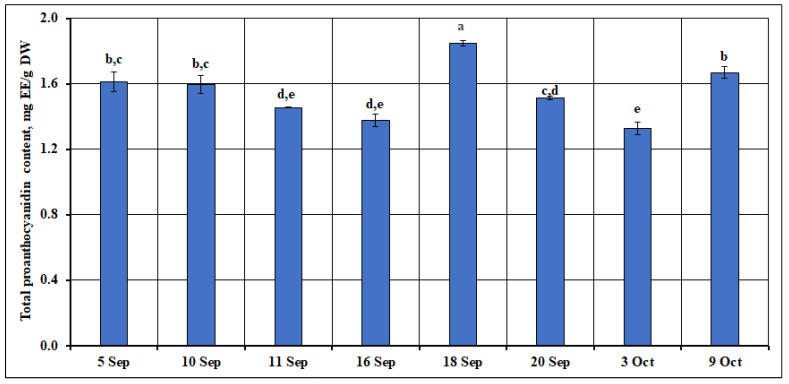
Variation in the total amount of proanthocyanidins in small cranberry fruit samples during the ripening period. Different letters indicate statistically significant differences in the total amount of proanthocyanidins between the *V. oxycoccos* fruit samples tested (*p* < 0.05).

The data presented by Jungfer et al. [21] indicate that proanthocyanidins accumulate in larger amounts in large cranberry fruit samples than in small cranberry fruit samples. Šedbarė et al. [22] reported that total amounts of proanthocyanidins found in small cranberry fruit samples collected in protected areas of Lithuania ranged from 0.92 mg EE/g to 3.04 mg EE/g. The highest total proanthocyanidin content (3.04 ± 0.14 mg EE/g) was found in fruit samples collected from mesotrophic-type wetland in the southern part of Lithuania (Žuvintas Biosphere Reserve) [22]. The levels found were 1.6–2.3 times higher than the total amount of proanthocyanidins found in our samples collected during the period of intense fruit ripening. The trends of the changes in the quantitative composition of proanthocyanidins found in the studies by Vvedenskaya et al. [16] and Wang et al. [23] differ from our results. These researchers indicate that the concentration of proanthocyanidins decreases most intensively at the beginning of small cranberry fruit ripening and increases later on in the ripening period [16,23]. During our study, the highest total amount of proanthocyanidins was determined in the middle of the intensive ripening period of the small cranberries. In order to assess the range of variation in the total proanthocyanidin content, a coefficient of variation (10.91%) was calculated, which shows a slight variation in the total proanthocyanidin content during the ripening period of the *V. oxycoccos* fruit. The variation in the total proanthocyanidin content in the small cranberry fruit samples during the ripening period is presented in Figure 1.

Hydroxycinnamic acids identified in medicinal plant materials have a wide range of biological effects: antioxidant, antimicrobial, anticancer, cardioprotective, hepatoprotective, anti-inflammatory, neuroprotective, antidiabetic, anti-aging, and other biological effects [24].

**Figure 2:**
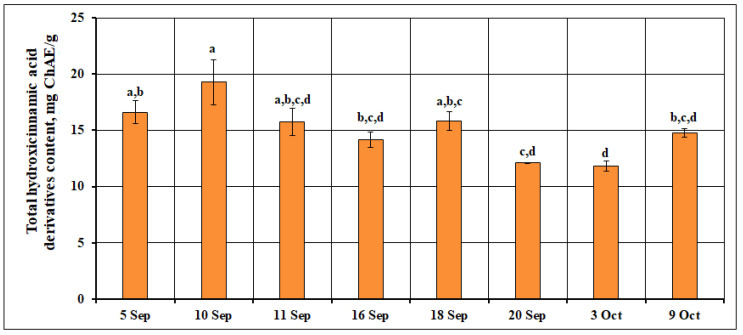
Variation in the total amount of hydroxycinnamic acid derivatives in small cranberry fruit samples during the ripening period. Different letters indicate statistically significant differences in the total amount of hydroxycinnamic acid derivatives between the *V. oxycoccos* fruit samples tested (*p* < 0.05).

The analysis using the spectrophotometric method showed that the total content of hydroxycinnamic acid derivatives in small cranberry fruit samples ranged from 11.85 mg ChAE (chlorogenic acid equivalent)/g to 19.27 mg ChAE/g. The calculated mean total amount of hydroxycinnamic acid derivatives in small cranberry fruit samples was 15.04 ± 2.43 mg ChAE/g. The highest total amounts of hydroxycinnamic acid derivatives (19.27 ± 2.01 mg ChAE/g) were found in samples of *V. oxycoccos* fruit collected at the beginning of the ripening period on September 10. At the beginning of fruit ripening, the biosynthetic pathways responsible for the production of hydroxycinnamic acid derivatives are highly active, as these compounds play a role in plant defense mechanisms and in regulating oxidative processes during early fruit development [25]. As ripening progresses, the concentrations of phenolic acids mostly decrease [26], and the plant’s metabolism shifts toward the synthesis of other secondary metabolites, such as anthocyanins and proanthocyanidins, which contribute to coloration and other ripening-related traits [27]. Therefore, the higher levels of hydroxycinnamic acid derivatives observed at the onset of ripening reflect their functional role in the early stages of fruit maturation and their subsequent decline as other compounds accumulate. As the fruit ripened, the total amount of hydroxycinnamic acid derivatives decreased. The lowest total amount of hydroxycinnamic acid derivatives (11.85 ± 0.44 mg ChAE/g) was detected in samples of small cranberry fruit collected at the end of the ripening period on October 3. The data from studies conducted by other researchers are consistent with our findings, which show a decrease in the amount of phenolic acids in small cranberry fruit samples during the ripening period. Oszmiański et al. [28] report that the content of phenolic acids decreased remarkably (by 12.3–36.5%) in fruit samples of different *V. macrocarpon* cultivars. The highest amounts of hydroxycinnamic acid derivatives at the beginning of the ripening period were also found in fruit samples of *Vaccinium corymbosum* L. [29]. The calculated coefficient of variation (16.12%) showed a slight variation in the total amount of hydroxycinnamic acid derivatives during the ripening of small cranberry fruits. The variation in the total amount of hydroxycinnamic acid derivatives during the ripening of *V. oxycoccos* fruits is presented in Figure 2.

In the fruit samples of *V. oxycoccos*, we identified and quantified chlorogenic acid, which belongs to the group of phenolic acids, as well as other compounds of the flavonol group: myricetin and its glycoside myricetin-3-galactoside, as well as quercetin and its glycosides hyperoside (quercetin-3-galactoside), isoquercitrin (quercetin-3-glucoside), guaijaverin (quercetin-3-arabinopyranoside), avicularin (quercetin-3-arabinofuranoside), and quercitrin (quercitrin-3-rhamnoside).

**Figure 3:**
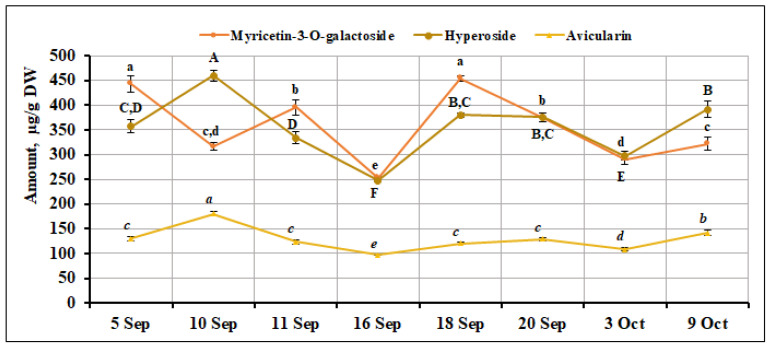
Variation in the quantitative composition of myricetin-3-O-galactoside, hyperoside, and avicularin in small cranberry fruit samples during the ripening period. Different letters (a–e for myricetin-3-O-galactoside, A–F for hyperoside, *a–e* for avicularin) indicate statistically significant differences in the content of these compounds between the *V. oxycoccos* fruit samples tested (*p* < 0.05).

The highest total amount of identified flavonols was determined in the samples of *V. oxycoccos* fruits collected on September 10 and September 18 (1232.84 ± 31.73 µg/g and 1159.85 ± 13.77 µg/g, respectively). The percentage of myricetin-3-galactoside content among the total amount of the identified flavonol compounds ranged from 25.66% to 39.41%, and the percentage of hyperoside content ranged from 31.76% to 37.27%. The percentage of avicularin in small cranberry fruit samples ranged from 10.42% to 14.64%. The highest content of this compound (180.51 ± 4.64 µg/g, *p* < 0.05) was found in fruit samples of *V. oxycoccos* collected on September 10, while the lowest content (96.89 ± 1.48 µg/g, *p* < 0.05) was found in fruit samples of *V. oxycoccos* collected on September 16. The percentage of hyperoside in small cranberry fruit samples collected in northern Lithuania (Kamanos State Strict Nature Reserve) and southern Lithuania (Žuvintas Biosphere Reserve) was found to be slightly higher (38.33%) than in the samples we analyzed. Meanwhile, the percentage of myricetin-3-galactoside (31.38%) and avicularin (12.37%) corresponded to the percentage of this compound in the fruit samples of *V. oxycoccos* collected in our study [22]. The percentage of avicularin (12.37%) in *V. oxycoccos* fruit samples collected in northern Lithuania (Kamanos State Strict Nature Reserve) and southern Lithuania (Žuvintas Biosphere Reserve) is in line with the results of our study (12.37%). Wang et al. [23] reported that the hyperoside content in *V. macrocarpon* fruit samples accounted for 31% to 46%, and myricetin-3-galactoside content accounted for 19% to 32% of the total quantified flavonol content. The highest amounts of myricetin-3-galactoside were detected in *V. oxycoccos* fruit samples collected on September 5 and September 18 (442.80 ± 15.61 µg/g and 454.67 ± 5.38 µg/g, respectively). The highest hyperoside content (459.49 ± 11.82 µg/g, *p* < 0.05) was found in small cranberry fruit samples collected on September 10. The lowest levels of myricetin-3-galactoside and hyperoside were found in *V. oxycoccos* fruit samples collected on September 16 (252.93 ± 3.87 µg/g and 247.70 ± 3.78 µg/g, respectively). The coefficient of variation for myricetin-3-O-galactoside was 20.37%, and for hyperoside, it was 17.97%. Oszmiański et al. [28] reported higher hyperoside (276.2–443.3 mg/100 g) and myricetin-3-O-galactoside (143.1–392.0 mg/100 g) contents in samples of large cranberry fruit. The flavonoid biosynthesis process can be influenced by temperature [30], soil water content [31], and other factors, which were not evaluated in this study. Urbstaite et al. [32] found higher levels of avicularin (293.79–613.80 µg/g) in *V. macrocarpon* fruit samples than those detected in small cranberry samples analyzed in this study. The percentage of hyperoside (34.9%), myricetin-3-O-galactoside (30.8%), and avicularin (12.3%) detected in small cranberry fruit samples collected in southern Lithuania (Čepkeliai State Strict Nature Reserve) were within the range of the results of our study [33]. The variation in the amount of myricetin-3-galactoside, hyperoside, and avicularin in small cranberry fruit samples during the ripening period is presented in Figure 3.

**Figure 4:**
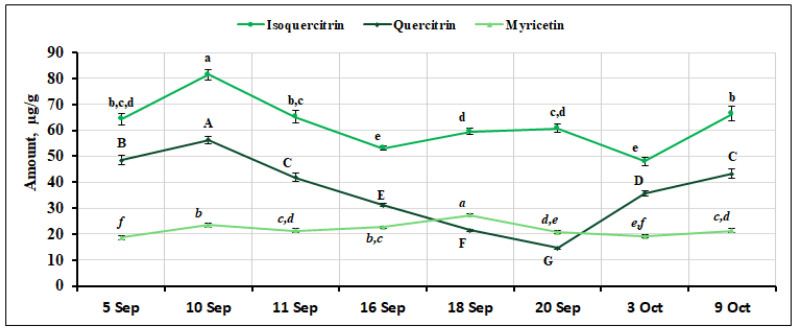
Variation in the amount of isoquercitrin, quercitrin, and myricetin in small cranberry fruit samples during the ripening period. Different letters (a–e for isoquercitrin, A–G for quercitrin, *a–f* for myricetin) indicate statistically significant differences in the content of these compounds between the *V. oxycoccos* fruit samples tested (*p* < 0.05).

We analyzed the quantitative composition of other flavonol compounds in small cranberry fruit samples. The percentage of isoquercitrin content varied from 5.26% to 6.91% of the total amount of the identified and quantified flavonol compounds. The highest content (81.44 ± 2.10 µg/g, *p* < 0.05) of isoquercitrin was found in fruit samples of *V. oxycoccos* collected on September 10, while the lowest content was found in fruit samples collected on September 16 and October 3 (53.07 ± 0.83 µg/g and 48.04 ± 1.57 µg/g, respectively, Figure 4). The percentage of quercitrin among the flavonol group compounds ranged from 1.39% to 4.57%. Its highest amount (56.32 ± 1.45 µg/g, *p* < 0.05) was detected in *V. oxycoccos* fruit samples collected on September 10, while the lowest amount (14.64 ± 0.38 µg/g, *p* < 0.05) was detected in fruit samples collected on September 20 (Figure 4). The coefficient of variation for quercitrin was calculated to be 37.82%, demonstrating a significant variation in the quantitative composition of this compound during the ripening period. The highest level of myricetin (27.34 ± 0.34 µg/g, *p* < 0.05) was detected on September 18, while the lowest levels were detected in fruit samples collected on September 5 and October 3 (18.59 ± 0.65 µg/g and 19.12 ± 0.63 µg/g, respectively, Figure 4). The percentage of myricetin content ranged from 1.65% to 2.95% among all the flavonol compounds detected. The coefficient of variation was calculated and showed a marginal variation of 12.63% for myricetin. The percentage of quercitrin (4.87%) in *V. oxycoccos* fruit samples collected in northern Lithuania (Kamanos State Strict Nature Reserve) and southern Lithuania (Žuvintas Biosphere Reserve) was slightly higher, and the percentage of myricetin (1.82%) was in line with the results of our study. In the fruit samples of small cranberries harvested in southern Lithuania (Čepkeliai State Strict Nature Reserve), the percentage of guaijaverin was slightly lower (4.7%), and the percentage of quercitrin and myricetin was higher (4.6% and 3.1%, respectively) than the percentage of these compounds in the fruit samples analyzed in our study [33]. Wang et al. [23] reported that in samples of large cranberries of different cultivars, the percentage of hyperoside was 31–46%, that of myricetin-3-galactoside was 19–32%, that of avicularin was 7–17%, and that of quercitrin was 7–14% of the total amount of flavonols found. Urbstaite et al. [32] reported higher levels of quercitrin (227.49–413.68 µg/g) in large cranberry fruit samples than those detected in *V. oxycoccos* fruit samples analyzed in this study. The variation in the amount of isoquercitrin, quercitrin, and myricetin in small cranberry fruit samples during the ripening period is presented in Figure 4.

**Figure 5:**
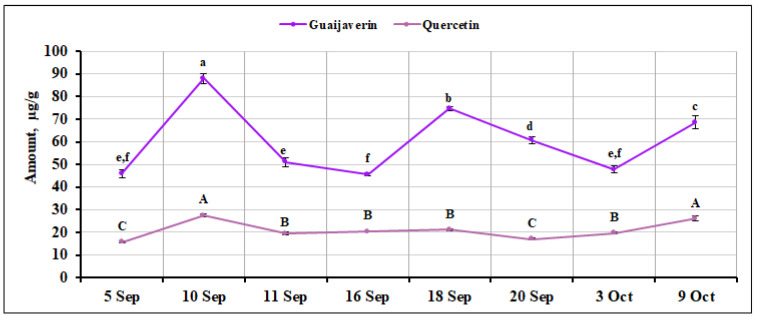
Variation in the quantitative composition of guaijaverin and quercetin in small cranberry fruit samples during the ripening period. Different letters (a–f for guaijaverin, A–C for quercetin) indicate statistically significant differences in the content of these compounds between the *V. oxycoccos* fruit samples tested (*p* < 0.05).

The percentage of the amount of guaijaverin among the flavonols detected ranged from 4.86% to 7.13%. The highest content (87.91 ± 2.26 µg/g, *p* < 0.05) was found in small cranberry fruit samples collected on September 10. The quantitative composition of guaijaverin varied quite significantly during the ripening of small cranberry fruit (Figure 5), with the calculated coefficient of variation being 25.84%. The highest levels of quercetin were found in *V. oxycoccos* fruit samples collected on September 10 and October 9 (27.47 ± 0.72 µg/g and 25.95 ± 1.10 µg/g, respectively). The lowest amounts of quercetin were found in small cranberry fruit samples collected on September 5 and September 20 (15.73 ± 0.55 µg/g and 17.02 ± 0.43 µg/g, respectively). The percentage of quercetin content found in the samples ranged from 1.40% to 2.64%. The amount of quercetin in the studied small cranberry fruit samples varied significantly, with the calculated coefficient of variation being 19.38%. Our results confirm those of Šedbarė et al. [22]. Survey data on small cranberries collected in northern Lithuania (Kamanos State Strict Nature Reserve) and southern Lithuania (Žuvintas Biosphere Reserve) show that quercetin accounted for 2.02% [22]. Šedbarė et al. [22] reported that the percentage of guaijaverin (4.57%) was slightly lower than that in the fruit samples analyzed in our study. In small cranberry fruit samples collected in southern Lithuania (Čepkeliai State Strict Nature Reserve), the percentage of quercetin was higher (4.4%) and the percentage of guaijaverin was slightly lower (4.7%) than those found in *V. oxycoccos* fruit samples collected during the ripening period that were analyzed in our study [33]. The variation in the quantitative composition of guaijaverin and quercetin is shown in Figure 5. The variation in the percentage composition of flavonol group compounds identified in all *V. oxycoccos* fruit samples is presented in the Supplementary Materials in Table S1.

**Figure 6:**
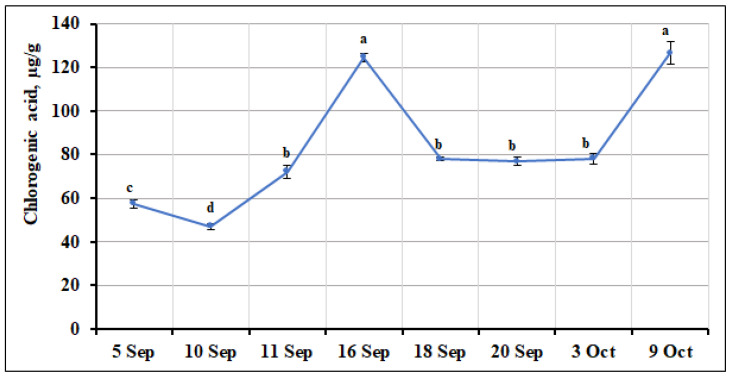
Variation in the quantitative composition of chlorogenic acid in small cranberry fruit samples during the ripening period. Different letters indicate statistically significant differences in the content of this compound between the *V. oxycoccos* fruit samples tested (*p* < 0.05).

Chlorogenic acid, a member of the group of phenolic acids, was also detected in small cranberry fruit samples. The highest amounts of this acid were found in small cranberry fruit samples collected on September 16 and October 9 (124.66 ± 1.90 µg/g and 126.55 ± 5.34 µg/g, respectively), while the lowest (47.04 ± 1.22 µg/g, *p* < 0.05) amounts were found in samples collected on September 10. The percentage of chlorogenic acid ranged from 3.68% to 13.93% of the total phenolic compounds identified and quantified. The coefficient of variation in the quantitative composition of chlorogenic acid in the samples collected during the ripening period of small cranberries showed a significant variation (coefficient of variation, CV = 34.80%) for this compound. Šedbarė et al. [22] reported that the chlorogenic acid content of *V. oxycoccos* fruit samples collected in northern Lithuania (Kamanos State Strict Nature Reserve) and southern Lithuania (Žuvintas Biosphere Reserve) ranged from 17 µg/g to 1224 µg/g. The highest amount of chlorogenic acid (1224 ± 41 µg/g) was found in fruit samples of small cranberries collected in the mesotrophic type wetland of northern Lithuania (Kamanos State Strict Nature Reserve) [22], which was 9.7 to 26 times higher than the chlorogenic acid content of the ripening fruit samples of *V. oxycoccos* analyzed in our study. Stobnicka and Gniewosz [34] tested fruit samples of *V. oxycoccos* extracted with different extractants and reported a higher content of chlorogenic acid (610–963 µg/g) than that found in the samples we tested. Higher amounts of chlorogenic acid (720.0–1296.2 µg/g) were also reported by Oszmiański et al. [17] in fruit samples of large cranberries. These results could be due to interspecies and intraspecies differences in the phytochemical composition of the plant organs, climatic conditions, soil geochemical composition, genetic [4,8,35,36], and other factors, which were not assessed in this study. The detailed data on the variation in the quantitative composition of chlorogenic acid in *V. oxycoccos* fruit samples during the ripening period are presented in Figure 6.

Studies on the phytochemical composition of small cranberry fruit in selected habitats to determine the qualitative and quantitative composition of flavonols, proanthocyanidins, proanthocyanidins, and hydroxycinnamic acid derivatives, as well as studies on the trends in the content of these compounds during the ripening period, are important for the preparation of high-quality small cranberry fruit raw material with a known chemical composition of bioactive compounds.

### 2.2. Determination of the Quantitative Composition of Anthocyanins

Anthocyanins found in berries, fruits, and vegetables are an important group of secondary metabolites found in plants. They protect plant organs from the harmful effects of UV radiation and adverse environmental conditions (frost, drought, oxidative processes, etc.) and give plant organs different colors [37]. Studies of the qualitative and quantitative composition of anthocyanins are important in order to assess the variation in anthocyanin composition of cranberry fruits during their development and ripening. Anthocyanin group compounds have hypoglycemic, antibacterial, anti-inflammatory, anticancer, memory-enhancing, antioxidant, and other biological properties [38].

UHPLC analysis of anthocyanins allowed the identification and quantification of 12 compounds in this group: 9 glycosides (delphinidin-3-O-galactoside, cyanidin-3-O-galactoside, cyanidin-3-O-glucoside, cyanidin-3-O-arabinoside, peonidin-3-O-galactoside, peonidin-3-O-glucoside, peonidin-3-O-arabinoside, malvidin-3-O-galactoside, and malvidin-3-O-glucoside) and 3 aglycones (anthocyanidins)—cyanidin, peonidin, and malvidin. The collection of small cranberry fruit samples during the ripening period was performed by using UHPLC according to the methodology described by Urbstaite et al. [32]. The highest total amount of identified anthocyanins (4096.79 ± 5.93 µg/g, *p* < 0.05) was determined in the samples of *V. oxycoccos* fruits collected on September 18.

**Figure 7:**
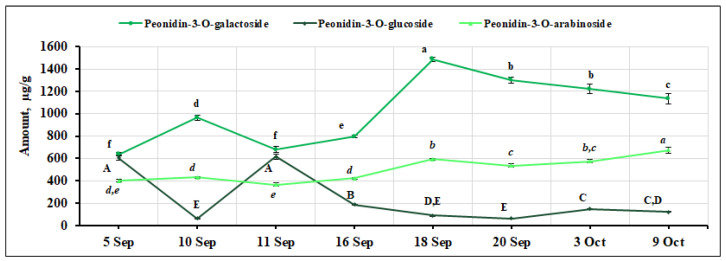
Variation in the quantitative composition of peonidin glycosides in small cranberry fruit samples during the ripening period. Different letters (a–f for peonidin-3-O-galactoside, A–E for peonidin-3-O-glucoside, *a*–*e* for peonidin-3-arabinoside) indicate statistically significant differences in the amount of anthocyanin compounds detected between the *V. oxycoccos* fruit samples tested (*p* < 0.05).

Three peonidin glycosides were identified and quantified in the small cranberry fruit samples tested: peonidin-3-O-galactoside, peonidin-3-O-glucoside, and peonidin-3-O-arabinoside. Peonidin-3-O-galactoside was the dominant compound among all the anthocyanin group compounds quantified. In 2011, Česonienė et al. published similar research data [39]. The calculated percentage of peonidin-3-O-galactoside ranged from 22.36% to 37.70%. The highest amount of glycoside (1486.04 ± 18.11 µg/g, *p* < 0.05) was detected in *V. oxycoccos* fruit samples collected on September 18, while the lowest amount was detected in fruit samples collected on September 5 and 11 (638.48 ± 15.73 µg/g and 680.76 ± 30.33 µg/g, respectively). The coefficient of variation (CV = 29.95%) showed a significant variation in peonidin-3-O-galactoside content. The percentage of peonidin-3-O-galactoside (26.4–30.4%) in small cranberry fruit samples collected in southern Lithuania (Čepkeliai State Strict Nature Reserve) reported by Šedbarė et al. [33] was in agreement with our results. Vorsa and Polashock [40] reported that the percentage of peonidin-3-O-galactoside in *V. oxycoccos* fruits (24.5%) was similar to that found in our samples. The percentage of peonidin-3-O-galactoside (29.15%) in cultured *V. oxycoccos* fruit samples found in a study by Česonienė et al. [39] is in line with the results obtained in our study. The evaluation of the quantitative composition of other peonidin glycosides in small cranberry fruit samples showed that other peonidin glycosides accounted for a lower percentage. The percentage of peonidin-3-O-arabinoside ranged from 13.94% to 20.38%. Its highest levels in *V. oxycoccos* fruit samples were found at the end of the ripening period, on October 9 (674.12 ± 28.42 µg/g, *p* < 0.05), and the lowest levels were found at the beginning of the ripening period, on September 5 and September 12 (402.22 ± 14.40 and 366.47 ± 14.47 µg/g, respectively). The peonidin-3-O-arabinoside content showed a moderate variation during the ripening of small cranberries with an estimated coefficient of variation of 21.97%. The percentage of peonidin-3-arabinoside (17.4–18.4%) in *V. oxycoccos* fruit samples collected in southern Lithuania (Čepkeliai State Strict Nature Reserve) was consistent with the results of our study [33]. The percentage of peonidin-3-O-arabinoside in small cranberry fruit reported by US scientists was 17.3% and is consistent with the percentage found in our samples [40]. The percentage of peonidin-3-arabinoside (19.64%) content in cultured *V. oxycoccos* fruit samples found by Česonienė et al. [39] is in agreement with the results of our study. The percentage of peonidin-3-O-glucoside content in small cranberry fruit samples collected during the ripening period ranged from 1.93% to 23.45%. The highest levels were found in small cranberry fruit samples collected on September 5 and September 11 (605.95 ± 21.82 µg/g and 616.42 ± 24.38 µg/g, respectively). The lowest levels of peonidin-3-O-glucoside were found in fruit samples of *V. oxycoccos* collected on September 10, September 18, and September 20 (63.00 ± 2.01 µg/g, 91.61 ± 1.10 µg/g, and 66.49 ± 1.65 µg/g, respectively). The calculated coefficient of variation (98.26%) demonstrates a very significant variation in the amount of peonidin-3-O-glucoside during the ripening of small cranberry fruits. The percentage of peonidin-3-glucoside (14.4%) in fruit samples of *V. oxycoccos* collected in southern Lithuania (Čepkeliai State Strict Nature Reserve) was within the range of our results [33]. The percentage of peonidin-3-O-glucoside in *V. oxycoccos* fruit reported by US scientists (4.7%) is in line with that found in our study [40]. Česonienė et al. [39] reported that the average percentage of peonidin-3-O-glucoside found in fruit samples of cultivated small cranberries of different cultivars was 6.21%. The variation in the peonidin glycoside content in small cranberry fruit samples during the ripening period is presented in Figure 7.

**Figure 8:**
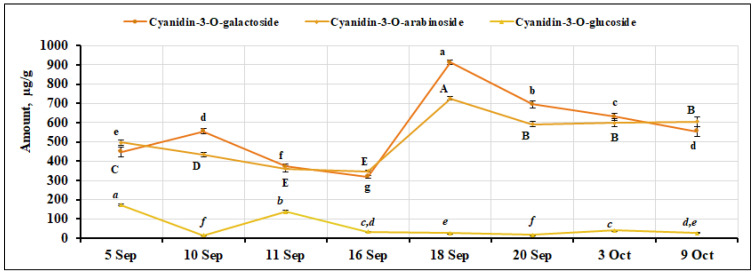
Variation in the quantitative composition of cyanidin glycosides in small cranberry fruit samples during the ripening period. Different letters (a–g for cyanidin-3-O-galactoside, A–E for cyanidin-3-O-arabinoside, *a–f* for cyanidin-3-O-glucoside) indicate statistically significant differences in the amount of anthocyanin compounds detected between the *V. oxycoccos* fruit samples tested (*p* < 0.05).

We qualitatively and quantitatively evaluated the cyanidin glycosides cyanidin-3-O-galactoside, cyanidin-3-O-arabinoside, and cyanidin-3-O-glucoside in *V. oxycoccos* fruit samples. Cyanidin-3-O-galactoside is the predominant anthocyanin among the quantitatively identified compounds, which is in line with the results obtained by other researchers [40,41]. Its percentage ranged from 13.84% to 22.26%. The highest content of cyanidin-3-O-galactoside (911.88 ± 10.71 µg/g, *p* < 0.05) was found in fruit samples collected on September 18, while the lowest content (318.59 ± 4.69 µg/g, *p* < 0.05) was found in fruit samples collected on September 16. The coefficient of variation showed a significant (33.86%) variation in cyanidin-3-O-galactoside content. The percentage of cyanidin-3-O-galactoside (16.2–20.1%) found in fruit samples of *V. oxycoccos* collected in southern Lithuania (Čepkeliai State Strict Nature Reserve) was consistent with the results of our study [33]. The percentage of cyanidin-3-O-galactoside in *V. oxycoccos* fruit samples reported by Vorsa and Polashock [40] (26.7%) was higher than that found in the small cranberry samples we examined. Česonienė et al. [39] reported that the percentage of cyanidin-3-O-galactoside in small cranberry samples was 20.44%, which is similar to that found in our samples. Other cyanidin glycosides were detected in lower amounts. The percentage of cyanidin-3-O-arabinoside ranged from 13.77% to 18.29%. The stages of fruit ripening cause variations in the chemical composition of small cranberries due to changes in metabolic activity during development. Early ripening stages are often characterized by the accumulation of certain phenolic compounds, such as phenolic acids, which serve protective and antioxidant roles [42]. As ripening progresses, the synthesis shifts toward other compounds like anthocyanins and proanthocyanidins, which contribute to fruit coloration, flavor, and defense [43]. Environmental factors and enzymatic activities also interact with these developmental processes, leading to dynamic changes in the chemical profile throughout ripening. Therefore, the chemical composition reflects the fruit’s physiological and biochemical status at each ripening stage [42]. Our study was limited solely to investigating the variation of biologically active compounds in cranberry fruits during the period of intensive fruit ripening; the assessment of the influence of enzymatic activity and environmental conditions was not the aim of this research. Its highest amount of cyanidin-3-O-arabinoside (726.36 ± 9.03 µg/g, *p* < 0.05) was detected in small cranberry fruit samples collected on September 18, while the lowest amounts were found in fruit samples collected on September 11 and 16 (362.04 ± 16.11 µg/g and 347.85 ± 5.17 µg/g, respectively). In our study, cyanidin-3-O-arabinoside showed a moderate variation of CV = 25.55% during the ripening of small cranberry fruit. The percentage of cyanidin-3-O-arabinoside (17.0–19.0%) found in fruit samples of *V. oxycoccos* collected in southern Lithuania (Čepkeliai State Strict Nature Reserve) was within the range of our results [33]. In the USA, researchers reported that the percentage of cyanidin-3-O-arabinoside in small cranberry fruit samples was 13.1% [40]. Česonienė et al. [39], who studied fruit samples of *V. oxycoccos* in Lithuania, reported a percentage of cyanidin-3-O-arabinoside (21.33%) that was slightly higher than that found in our samples. The percentage of cyanidin-3-O-glucoside was the lowest of all the cyanidin glycosides detected, ranging from 0.49% to 6.02%. The highest level of cyanidin-3-O-glucoside was detected in fruit samples collected on September 5 (171.93 ± 4.68 µg/g, *p* < 0.05), while the lowest levels of cyanidin-3-O-glucoside were detected in fruit samples collected on September 10 and 20 (15.60 ± 0.45 µg/g and 16.91 ± 0.45 µg/g, respectively). The quantitative composition of cyanidin-3-O-glucoside varied over a wide range, with an estimated CV = 102.80%. The percentage of cyanidin-3-O-glucoside (3.9%) found in fruit samples of *V. oxycoccos* collected in southern Lithuania (Čepkeliai State Strict Nature Reserve) was in line with the results of our study [33]. The percentage of cyanidin-3-O-glucoside content (3.23%) reported by Česonienė et al. [39] is consistent with our results. The variation in the quantitative composition of cyanidin glycosides in small cranberry fruit samples during the ripening period is shown in Figure 8.

**Figure 9:**
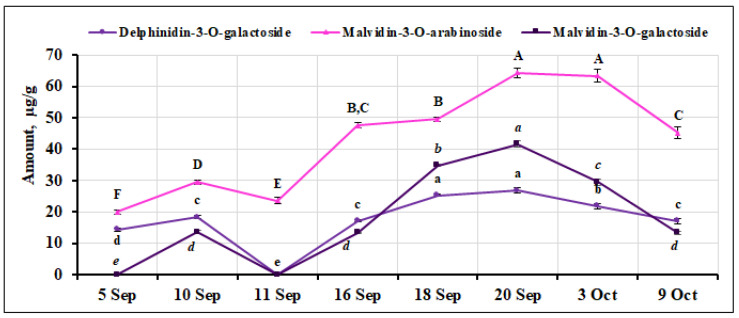
Variation in the quantitative composition of malvidin glycosides and delphinidin-3-O-galactoside in small cranberry fruit samples during the ripening period. Different letters (a–e for delphinidin-3-O-galactoside, A–F for malvidin-3-O-arabinoside, *a–e* for malvidin-3-O-galactoside) indicate statistically significant differences in the amount of anthocyanin compounds detected between the *V. oxycoccos* fruit samples tested (*p* < 0.05).

Two malvidin glycosides, malvidin-3-O-arabinoside and malvidin-3-O-galactoside, were identified and quantified in small cranberry fruit samples. The percentage of malvidin-3-O-arabinoside in *V. oxycoccos* fruit samples during the ripening period ranged from 0.70% to 2.07%. The highest amounts of this compound were found in fruit samples collected on September 20 and October 3 (64.32 ± 1.60 µg/g and 63.35 ± 2.06 µg/g, respectively), while the lowest amount (19.97 ± 0.70 µg/g, *p* < 0.05) was found in samples collected on September 5. The quantitative composition of malvidin-3-O-arabinoside varied significantly during the ripening of small cranberry fruit (CV = 39.64%). The percentage of malvidin-3-galactoside ranged from 0.40% to 1.21% during the ripening period. The highest amount of this compound (41.60 ± 0.93 µg/g, *p* < 0.05) was detected in the fruit samples collected on September 20, while the lowest amounts were found in the fruit samples collected on September 10, September 16, and October 9 (13.62 ± 0.42 µg/g, 13.30 ± 0.25 µg/g, and 13.36 ± 0.57 µg/g, respectively). The quantitative composition of malvidin-3-galactoside varied widely during the ripening of the fruit, with an estimated CV = 51.65%. Delphinidin-3-O-galactoside was also identified and quantified in small cranberry samples. In our study, the percentage of this compound ranged from 0.50% to 0.78%. The highest levels of delphinidin-3-O-galactoside were found in fruit samples collected on September 18 and 20 (25.32 ± 0.30 µg/g and 26.76 ± 0.82 µg/g, respectively), while the lowest level (14.40 ± 0.47 µg/g) was found in fruit samples collected on September 5. The coefficient of variation was calculated and showed a mean variation (CV = 23.01%) for delphinidin-3-O-galactoside. Šedbarė et al. [44], who studied the fruit of large cranberries, reported a percentage of malvidin-3-O-galactoside (0.45%) that was similar to that found in our samples, with the percentage of malvidin-3-O-arabinoside (0.61%) being slightly lower and the percentage of delphinidin-3-O-galactoside (2.04%) being higher than that found in the samples we studied. The variation in the quantitative composition of malvidin glycosides and delphinidin-3-O-galactoside in small cranberry fruit samples during the ripening period is shown in Figure 9.

**Figure 10:**
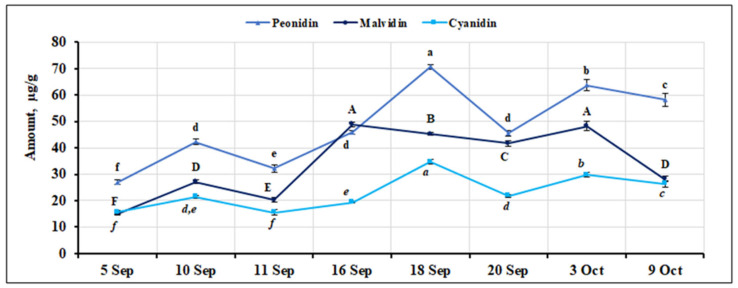
Variation in the quantitative composition of anthocyanidins in small cranberry fruit samples during the ripening period. Different letters (a–f for peonidin, A–F for malvidin, *a–f* for cyanidin) indicate statistically significant differences in the content of anthocyanidin group compounds between the *V. oxycoccos* fruit samples tested (*p* < 0.05).

Three anthocyanin aglycones—anthocyanidins peonidin, malvidin, and cyanidin—were identified and quantified in small cranberry fruit samples. The percentage of peonidin in *V. oxycoccos* fruit samples during the ripening period ranged from 0.95% to 2.00%. The highest amount of this aglycone (70.64 ± 0.84 µg/g, *p* < 0.05) was detected in fruit samples collected on September 18, while the lowest amount (27.01 ± 0.95 µg/g, *p* < 0.05) was detected in fruit samples collected on September 5. Peonidin content varied significantly during the ripening period, with an estimated CV = 31.35%. The percentage of malvidin in *V. oxycoccos* fruit samples ranged from 0.53% to 2.12% during the ripening period. The highest amounts of this compound were found in fruit samples collected on September 16 and October 3 (48.85 ± 0.75 µg/g and 48.30 ± 1.92 µg/g, respectively), while the lowest amount was found in samples collected on September 5 (15.14 ± 0.53 µg/g, *p* < 0.05). The quantitative composition of malvidin in small cranberry fruit samples during the ripening period varied quite widely, with an estimated CV = 38.66%. The percentage of cyanidin in *V. oxycoccos* fruit samples during the ripening period ranged from 0.55% to 0.86%. The highest amounts of this aglycone (34.72 ± 0.85 µg/g, *p* < 0.05) were found in fruit samples collected on September 18, while the lowest amounts were found in fruit samples collected on September 5 and September 11 (15.60 ± 0.58 µg/g and 15.51 ± 0.96 µg/g, respectively. The variation in the quantitative composition of anthocyanidins identified and quantified in small cranberry fruit samples during the ripening period is shown in Figure 10. The variation in the percentage composition of all anthocyanins identified in all *V. oxycoccos* fruit samples is presented in the Supplementary Materials in Table S2.

We chose a site where the samples were collected and where it was appropriate to determine the qualitative and quantitative composition of bioactive compounds. The results of the study showed an increasing trend in the content of most of the identified compounds of the anthocyanin group during the ripening of small cranberry fruit, which provides insight into the quantitative changes of individual compounds and allows for the timing of small cranberry fruit harvesting when the concentration of anthocyanins is highest. Chemical composition studies on small cranberry fruit samples allow for the qualitative and quantitative assessment of the composition of individual anthocyanins and for the targeted preparation of small cranberry raw material with the highest content of anthocyanins, which are natural pigments with strong antioxidant activity.

### 2.3. Determination of the Quantitative Composition of Triterpene Compounds

The outer surface of the fruit peel is covered with a waxy layer that protects the fruit from water evaporation, temperature changes, UV radiation, and microorganisms [45]. The waxy layer of cranberry berries has been found to contain triterpene compounds [46], which have a biological effect. Triterpene compounds have anti-inflammatory, antibacterial, antiviral, hepatoprotective, gastroprotective, cardioprotective, hypolipidemic, immunoregulatory, antiatherosclerotic, anticancer, and antioxidant pharmacological activities [47].

Using UHPLC, nine compounds of the triterpene group were identified and quantified in small cranberry fruit samples: maslinic acid, corosolic acid, oleanolic acid, ursolic acid, campesterol, α-amyrin, β-sitosterol, and squalene. The highest total amount of identified triterpene compounds (8248.46 ± 125.60 µg/g, *p* < 0.05) was determined in the samples of *V. oxycoccos* fruits collected on September 16.

**Figure 11:**
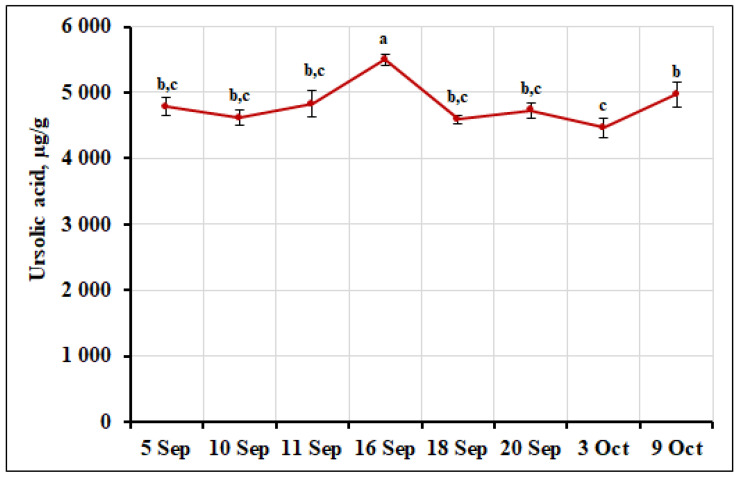
Variation in the amount of ursolic acid in small cranberry fruit samples during the ripening period. Different letters indicate statistically significant differences in ursolic acid content between the *V. oxycoccos* fruit samples tested (*p* < 0.05).

Four triterpenic acids were detected in small cranberry fruit samples: maslinic acid, corosolic acid, oleanolic acid, and ursolic acid. Ursolic acid was the predominant acid in the fruit samples of *V. oxycoccos* collected during the ripening period. Data published by other researchers confirm our results, showing that ursolic acid was the most abundant triterpene compound in small cranberry fruit samples [45]. The percentage of ursolic acid ranged from 63.83% to 67.49% of the total amount of triterpenic compounds detected in the samples. Its highest amount (5495.43 ± 83.68 µg/g, *p* < 0.05) was detected in fruit samples of *V. oxycoccos* collected on September 16. The coefficient of variation (6.61%) showed a slight variation in ursolic acid in the fruit samples tested. The variation in triterpene compounds content during cranberry fruit ripening is influenced by multiple factors, including the developmental stage of the fruit [17], which affects biosynthetic activity; the enzymatic processes involved in triterpenoid synthesis that fluctuate throughout ripening; genetic differences among cultivars; and various environmental conditions such as climate and other factors [48]. Additionally, biotic and abiotic stresses can modulate triterpenoid production as part of the plant’s defense mechanisms [49]. Together, these factors contribute to the dynamic changes observed in triterpene compounds levels during the ripening process. The percentage of ursolic acid was higher in fruit samples of *V. oxycoccos* collected from northern Lithuania (Kamanos State Strict Nature Reserve) and southern Lithuania (Žuvintas Biosphere Reserve), accounting for 76.24% of the total amount of triterpene compounds quantified [22]. A higher percentage of ursolic acid (76.8%) than in our study was also found in small cranberry fruit samples collected in southern Lithuania (Čepkeliai State Strict Nature Reserve) [33]. Zhang et al. [50] analyzed cranberry fruit samples and found 65.9 mg/100 g of ursolic acid. The variation in the amount of ursolic acid in small cranberry fruit samples collected during the ripening period is presented in Figure 11.

**Figure 12:**
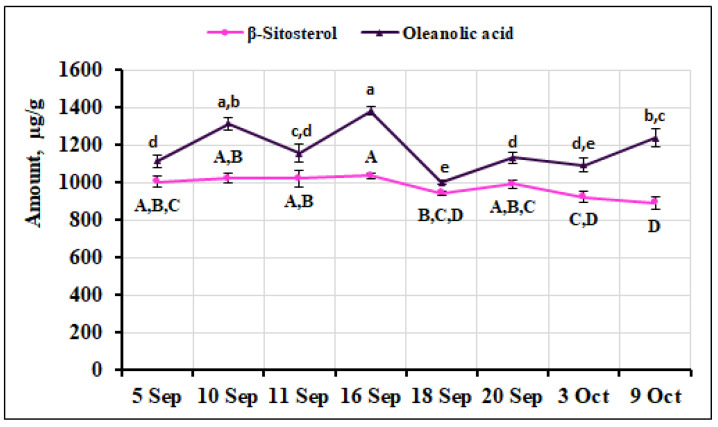
Variation in the quantitative composition of β-sitosterol and oleanolic acid in small cranberry fruit samples during the ripening period. Different letters (a–e for oleanolic acid, A–D for β-sitosterol) indicate statistically significant differences in the content of these compounds between the *V. oxycoccos* fruit samples tested (*p* < 0.05).

The percentage of oleanolic acid in our fruit samples ranged from 14.68% to 18.16%. The highest amounts were found in fruit samples collected on September 10 and 16 (1314.54 ± 32.95 µg/g and 1381.18 ± 21.03 µg/g, respectively). The lowest amounts were found in fruit samples collected on September 18 and October 3 (997.99 ± 11.81 µg/g and 1093.18 ± 36.29 µg/g, respectively). The calculated coefficient of variation (10.62%) reflects a slight variation in the oleanolic acid content of the fruit samples. The percentage of oleanolic acid (17.44%) in small cranberry fruits collected in northern Lithuania (Kamanos State Strict Nature Reserve) and southern Lithuania (Žuvintas Biosphere Reserve) reported by Šedbarė et al. [22] was in line with the results of our study. The percentage of oleanolic acid (17.12%) found in samples of small cranberries collected in southern Lithuania (Čepkeliai State Strict Nature Reserve) was within the range of our results [33]. The percentage of β-sitosterol in the total amount of triterpene compounds detected in small cranberry fruits ranged from 11.97% to 14.11%. The highest amount of β-sitosterol (1034.65 ± 15.75 µg/g) was detected in small cranberry fruit samples collected on September 16, but it was not statistically significantly different from the amount of this compound in the fruit samples collected on September 5, September 10, September 11, or September 20 (1002.39 ± 30.20 µg/g, 1020.98 ± 25.60 µg/g, 1020.23 ± 42.85 µg/g, and 990.48± 23.74 µg/g, respectively). The lowest amount of β-sitosterol (890.34 ± 33.83 µg/g) was detected in small cranberry fruit samples collected on October 9, but it was not statistically significantly different from the amount of β-sitosterol detected in the fruit samples collected on September 18 or October 3 (943.54 ± 11.17 µg/g and 922.52 ± 30.62 µg/g, respectively). The quantitative composition of β-sitosterol in small cranberry fruit samples during the ripening period showed the least variation of all the phenolic compounds identified and quantified, with an estimated coefficient of variation of 5.40%. Wu et al. [51] indicate that β-sitosterol is the major compound in the phytosterol group of cranberry fruits, which is consistent with our results. Sedbarė et al. [52] reported slightly higher β-sitosterol content (1068.3 ± 16.02 µg/g) in small cranberry fruit samples than that found in our samples. The variation in the quantitative composition of β-sitosterol and oleanolic acid in samples of small cranberry fruit collected during the ripening period is shown in Figure 12.

**Figure 13:**
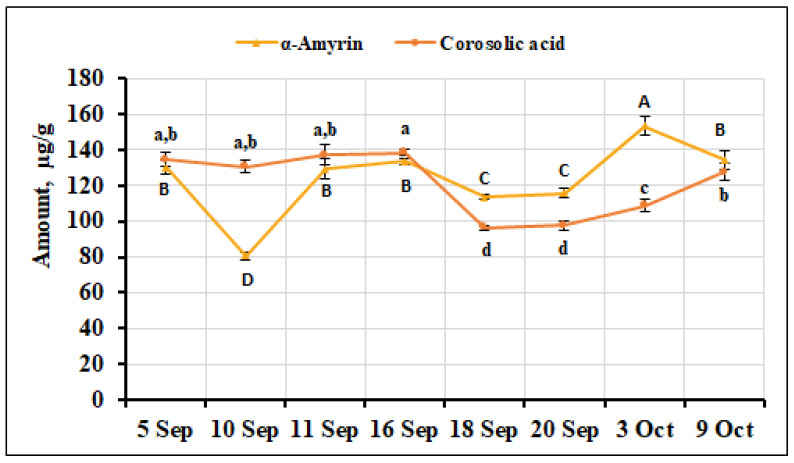
Variation in the quantitative composition of α-amyrin and corosolic acid in small cranberry fruit samples during the ripening period. Different letters (a–d for corosolic acid, A–D for α-amyrin) indicate statistically significant differences in the content of these compounds between the *V. oxycoccos* fruit samples tested (*p* < 0.05).

The percentage of corosolic acid in the small cranberry fruit samples ranged from 1.37% to 1.87%. Its highest amount (138.56 ± 2.11 µg/g) was detected in *V. oxycoccos* fruit samples collected on September 16, but it was not statistically significantly different from the levels of corosolic acid detected in fruit samples collected on September 5, September 10, or September 11 (134.52 ± 4.07 µg/g, 130.61 ± 3.29 µg/g, and 137.07 ± 5.76 µg/g, respectively). The lowest amounts of corosolic acid were found in small cranberry fruit samples collected on September 18 and September 20 (96.42 ± 1.14 µg/g and 97.62 ± 2.34 µg/g, respectively). The calculated coefficient of variation (14.56%) reflects a slight variation in the quantitative composition of this acid in the small cranberry samples analyzed. The percentage of α-amyrin among the total amount of triterpene compounds detected ranged from 1.11% to 2.26%. The highest amount of this compound (153.24 ± 5.09 µg/g, *p* < 0.05) was detected in the fruit samples collected on October 3, while the lowest amount (80.57 ± 2.02 µg/g, *p* < 0.05) was detected in the fruit samples collected on September 10. The quantitative composition of α-amyrin in the small cranberry fruit samples showed a slight variation during the ripening period, with an estimated coefficient of variation of 17.20%. β-Amyrin was only quantified in the small cranberry fruit samples collected on October 9, and its amount was found to be 24.76 ± 0.94 µg/g. The percentage corosolic acid (7%) in the fruit samples of *V. oxycoccos* collected in northern Lithuania (Kamanos State Strict Nature Reserve) was significantly higher than that found in our samples [22]. The percentage of corosolic acid (3.2%) found in samples of small cranberries collected in southern Lithuania (Čepkeliai State Strict Nature Reserve) was higher than that found in our study [33]. The percentage of α-amyrin content (1.98%) in *V. oxycoccos* fruit samples collected in northern Lithuania (Kamanos State Strict Nature Reserve) and southern Lithuania (Žuvintas Biosphere Reserve) was within the range of our results [22]. The percentage of α-amyrin (1.65%) found in fruit samples of small cranberries collected in southern Lithuania (Čepkeliai State Strict Nature Reserve) was in line with the results of our study [33]. Sedbarė et al. [52] found a lower amount of α-amyrin (57.1 ± 0.86 μg/g) in *V. oxycoccos* fruit samples. The variation in α-amyrin and corosolic acid contents of small cranberry fruit samples collected during the ripening period is presented in Figure 13.

**Figure 14:**
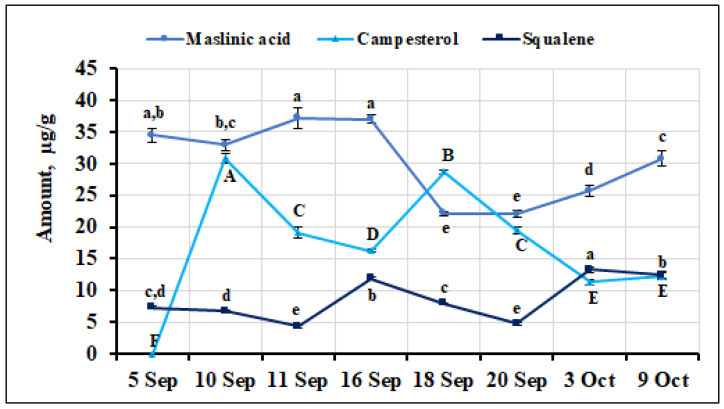
Variation in the quantitative composition of maslinic acid, campesterol, and squalene in small cranberry fruit samples during the ripening period. Different letters (a–e for maslinic acid, A–F for campesterol, *a*–*e* for squalene) indicate statistically significant differences in the content of these compounds between the *V. oxycoccos* fruit samples tested (*p* < 0.05).

The percentage of maslinic acid of the total amount of triterpene compounds detected in *V. oxycoccos* fruit samples during the ripening period ranged from 0.31% to 0.51%. The highest amount of maslinic acid (37.22 ± 1.61 µg/g) was found in fruit samples collected on September 11, but it was not statistically significantly different from that found in samples collected on September 5 or September 16 (34.49 ± 1.13 µg/g and 37.04 ± 0.71 µg/g, respectively). The lowest amount of maslinic acid was found in *V. oxycoccos* fruit samples collected on September 18 and 20 (22.09 ± 0.34 µg/g and 22.05 ± 0.53 µg/g, respectively). The quantitative composition of maslinic acid in small cranberry fruit samples collected during the ripening period showed the greatest variation of all the triterpenic acids detected, with a coefficient of variation calculated at 20.69%. The percentage of maslinic acid (2%) in the fruit samples of *V. oxycoccos* collected in northern Lithuania (Kamanos State Strict Nature Reserve) was significantly higher than that found in our samples [22]. The percentage of maslinic acid (0.83%) found in samples of small cranberries collected in southern Lithuania (Čepkeliai State Strict Nature Reserve) was higher than that found in our study [33]. The amount of campesterol detected in the small cranberry samples tested was significantly lower, with percentages ranging from 0.16% to 0.43% of the total amount of triterpene compounds quantified. The highest amount of campesterol (30.92 ± 0.78 µg/g, *p* < 0.05) was found in fruit samples collected on September 10, while the lowest amount was found in fruit samples collected on October 3 and 9 (11.28 ± 0.38 µg/g and 12.24 ± 0.46 µg/g, respectively). Small cranberry fruit samples collected during the ripening period showed the presence of a non-cyclic triterpene compound, squalene. Its percentage varied from 0.06% to 0.20% among all the identified and quantified triterpene compounds. The highest amount of squalene (13.29 ± 0.44 µg/g, *p* < 0.05) was detected in fruit samples collected on October 3, while the lowest amount was detected in fruit samples collected on September 11 and September 20 (4.32 ± 0.19 µg/g and 4.73 ± 0.12 µg/g, respectively). The quantitative composition of squalene in small cranberry fruit samples collected during the ripening period showed the greatest variation of all the triterpene compounds detected, with a calculated coefficient of variation of 40.93%, reflecting a significant variation in the content of this compound among the samples analyzed. Sedbare et al. [52] reported higher squalene content (48.6 ± 0.73 µg/g) in small cranberry fruit samples than that found in our samples. The variation in the amount of maslinic acid, campesterol, and squalene in small cranberry fruit samples collected during the ripening period is shown in Figure 14. The variation in the percentage composition of all triterpene compounds identified in all *V. oxycoccos* fruit samples is presented in the Supplementary Materials in Table S3.

Small cranberry fruit plant material accumulates triterpene compounds with a wide range of biologically active properties, and therefore, studies on the qualitative and quantitative composition of these compounds are relevant. The triterpene compounds detected in small cranberry fruit can be used as markers for the detection of triterpenes in *V. oxycoccos* fruit samples during the ripening period, as well as in the preparation of small cranberry fruit. Studies on the chemical composition of the fruit allow for the provision of high-quality plant material with a known qualitative and quantitative composition of triterpene compounds.

### 2.4. Determination of Antioxidant Activity of V. oxycoccos Fruit Extracts

Recently, studies have been carried out to identify compounds in plant matrices with strong antioxidant effects. Antioxidants inhibit inflammatory processes, slow down aging, and protect against oxidative stress [53]. For these reasons, it is worthwhile to investigate the variation in the quantitative composition of phenolic and triterpene compounds during the ripening of small cranberries and to assess the dynamics of the variation of antiradical and reductive activities during ripening in order to determine the fruit harvesting time at which the selected small cranberry fruit extracts exhibit the strongest antioxidant activity in vitro. It is thus of interest to assess the correlation between the amounts of bioactive compounds detected and the in vitro antioxidant activity.

The in vitro antiradical activity of *V. oxycoccos* fruit sample extracts ranged from 75.16 µmol TE (Trolox equivalents)/g to 95.25 µmol TE/g. The mean in vitro antiradical activity of all the extracts tested was found to be 83.71 ± 7.15 µmol TE/g. The strongest in vitro antiradical activity was observed for the extracts of small cranberry fruit collected on September 10 and September 18 (95.25 ± 1.15 µmol TE/g and 92.16 ± 0.20 µmol TE/g, respectively). The weakest in vitro antiradical activity (75.16 ± 2.42 µmol TE/g) was found in extracts of small cranberry fruit collected on September 16, but it was not statistically significantly different from that found in extracts of small cranberry fruit collected on September 11, September 20, or October 3 (79.98 ± 0.77 µmol TE/g, 76.31 ± 3.51 µmol TE/g, and 81.34 ± 2.34 µmol TE/g, respectively). The coefficient of variation, which reflects the range of variation in the in vitro antiradical activity of the tested extracts, was found to be insignificant (CV = 8.54%). The in vitro antiradical activity of the extracts of small cranberry fruit collected from different locations of Lithuania, as assessed by the ABTS (2,2′-azino-bis(3-ethylbenzothiazoline-6-sulfonic acid) assay, varied over a wider range (CV = 18.86%), from 37.38 μmol TE/g to 141.28 μmol TE/g [54].

**Figure 15:**
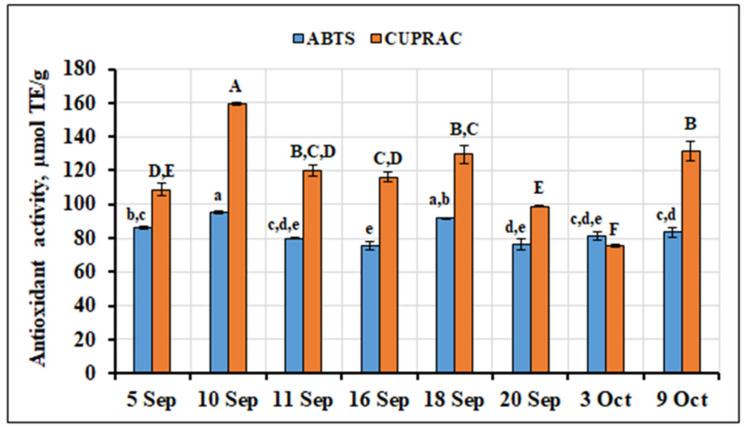
Variation in the in vitro antioxidant activity of the extracts of *V. oxycoccos* fruit samples collected during the ripening period. Different letters (a–e for ABTS, A–F for CUPRAC) indicate statistically significant differences between the antioxidant activity estimates of the tested small cranberry fruit sample extracts (*p* < 0.05).

The in vitro reducing activity of the extracts of small cranberry samples collected during the ripening period was found to range from 75.33 µmol TE/g to 159.26 µmol TE/g. The mean calculated reducing activity of all *V. oxycoccos* fruit sample extracts tested was 117.45 ± 24.82 µmol TE/g. The strongest in vitro reducing activity (159.26 ± 0.77 µmol TE/g, *p* < 0.05) was observed in extracts of small cranberry fruit collected on September 10, and the weakest (75.33 ± 0.78 µmol TE/g, *p* < 0.05) was found in extracts of fruit collected on October 3. The calculated coefficient of variation showed a moderate variation (CV = 21.13%) in the in vitro reducing activity of the extracts of *V. oxycoccos* fruit samples collected during the ripening period. The in vitro reducing activity of fruit extracts of large cranberries grown in Lithuania, as assessed by the CUPRAC (CUPric Reducing Antioxidant Capacity) method, was stronger (215.15–493.87 μmol TE/g) than that observed in the present study. Namiesnik et al. [55], who investigated the in vitro reducing activity of large cranberry fruit extracts using the CUPRAC methodology, reported a weaker activity (49.38 ± 4.4 µM TE/g) than what we observed in our tested small cranberry fruit extracts. The variation in the in vitro antiradical and reducing activities of the extracts of small cranberry fruit collected in natural habitats during the ripening period is presented in Figure 15.

A statistical correlation analysis was performed to assess the relationship between the quantitative composition of anthocyanins, proanthocyanidins, hydroxycinnamic acid derivatives, flavonols, chlorogenic acid, and triterpene compounds collected from *V. oxycoccos* fruit samples during the ripening period and the in vitro antiradical and reducing activity of *V. oxycoccos* fruit extracts.

Statistical analysis showed a strong correlation between the total amount of hydroxycinnamic acid derivatives in small cranberry fruit samples and the in vitro reducing and antiradical activity of their extracts (r = 0.858, *p* < 0.01 and r = 0.771, *p* < 0.05, respectively). There was a strong correlation (r = 0.711, *p* < 0.01) between the total amount of proanthocyanidins in *V. oxycoccos* fruit samples and the in vitro antiradical activity of their extracts. A moderate correlation (r = 0.609, *p* < 0.05) was found between the total amount of proanthocyanidins in the tested small cranberry fruit samples and the in vitro reducing activity of their extracts.

In the group of the identified flavonol compounds, the strongest positive correlation between individual compounds was found between the amounts of isoquercitrin (r = 0.844, *p* < 0.01) and guaijaverin (r = 0.769, *p* < 0.05) and the in vitro reducing activity assessed by the CUPRAC method. The levels of avicularin and quercitrin detected in small cranberry fruit samples correlated strongly with the in vitro reducing activity of the tested sample extracts (r = 0.721, *p* < 0.05 and r = 0.731, *p* < 0.05, respectively). The amount of guaijaverin and hyperoside in small cranberry fruit samples correlated strongly with the in vitro antiradical activity assessed by the ABTS assay (r = 0.754, *p* < 0.05 and r = 0.747, *p* < 0.05, respectively). All the results of the Pearson’s correlation analysis we performed are presented in the Supplementary Material (Table S4). In our previous study, we found a moderately strong correlation between the ABTS-assessed in vitro antiradical activity of *V. oxycoccos* fruit sample extracts and the amounts of myricetin-3-O-galactoside and hyperoside (r = 0.660, *p* < 0.01 and r = 0.606, *p* < 0.01, respectively) [54].

Oszmiański et al. [17] reported a positive moderate correlation between the amount of flavonols and the total content of all the identified phenolic compounds in large cranberry fruit extracts and the in vitro antiradical activity of these extracts assessed by the ABTS assay (r = 0.646 and r = 0.508, accordingly), a strong positive correlation (r = 0.728) between the flavonol content and the in vitro reducing activity assessed by the FRAP method, and a moderate positive correlation (r = 0.591) between the total content of phenolic compounds and the in vitro reducing activity. These authors reported a strong positive correlation between the in vitro antiradical and reducing activity of large cranberry fruit extracts and the total amount of triterpene compounds (r = 0.852 and r = 0.736, respectively), as well as a moderate positive correlation between the amount of anthocyanins and the in vitro antiradical and reducing activity of these extracts (r = 0.675 and r = 0.614, respectively) [17].

Small cranberry fruits are a valuable plant material accumulating flavonols, phenolic acids, proanthocyanidins, anthocyanins, and triterpene compounds. In plant matrices, it is often not possible to attribute biological effects to a specific group of compounds or to a specific compound, as the complex of compounds they accumulate act synergistically [56]. As the quantitative composition of the biologically active compounds of *V. oxycoccos* fruits changes during the ripening period, so does the antioxidant activity of small cranberry fruit extracts, and its evaluation is important to ensure the quality of the *V. oxycoccos* fruits and the biological effects of the preparations made from them.

## 3. Materials and Methods

### 3.1. Plant Material

The samples of small cranberry fruits (200 g each) growing under Lithuanian climatic conditions used in this study were hand-picked in the village of Janoniai, Molėtai district, from a wetland near Lake Pagulbis (55°22′59.0″ N 25°13′58.4″ E), during September–October 2021. During the collection of *V. oxycoccos* fruit samples in the Molėtai district, where the Pagulbis Lake bog is located, the average air temperature was 10.6 °C in September and 7.6 °C in October. The average precipitation in Molėtai was 37.0 mm in September and 31.9 mm in October. *V. oxycoccos* fruit samples were frozen at −20 °C temperature and stored until lyophilization. Small cranberry fruits were lyophilized using the technique described by Šedbarė et al. [22]. All the presented data were recalculated for dry weight.

### 3.2. Extraction Procedure

The extraction of biologically active compounds from *V. oxycoccos* fruit samples was performed by applying a technique described by Liaudanskas et al. [57], using a 2% (*v*/*v*) solution of hydrogen chloride in 70% (*v*/*v*) ethanol as an extractant.

### 3.3. Spectrophotometric Analysis

All the spectrophotometric measurements were carried out with an i3 UV-VIS spectrophotometer (Hanon instrument Co., Ltd., Jinan, China).

#### 3.3.1. Determination of the Total Amount of Proanthocyanidins and Hydroxycinnamic Acid Derivatives

In the first stage of our study, we applied spectrophotometry to assess the variation in the quantitative composition of the different groups of phenolic compounds—proanthocyanidins and hydroxycinnamic acid derivatives—in small cranberry fruit samples. The total amount of proanthocyanidins in the extracts of *V. oxycoccos* fruit samples was evaluated by the DMCA (4-(dimethylamino)cinnamaldehyde) assay according to the methodology described by Šedbarė et al. [22] with minor modifications. A 50 μL measure of small cranberry fruit extract was used for the analysis. The total amount of proanthocyanidins was expressed as (–)-epicatechin equivalents (EEs) in mg/g of the small cranberry fruit samples. Meanwhile, the total amount of hydroxycinnamic acid derivatives in the extracts of *V. oxycoccos* fruit samples was determined according to the technique described by Zymonė et al. [58] and expressed as chlorogenic acid equivalents (ChAEs) in mg/g of the *V. oxycoccos* fruit samples.

#### 3.3.2. Determination of Antioxidant Activity In Vitro

The ABTS and CUPRAC assays were performed using the techniques described by Balciunaitiene et al. [59]. The in vitro antioxidant activity of the tested extracts was expressed as Trolox equivalents (TE) in µmol/g of the *V. oxycoccos* fruit samples.

### 3.4. Chromatographic Methods

The evaluation of anthocyanin content in the extracts of *V. oxycoccos* fruit samples was performed according to the technique described by Vilkickyte et al. [60]. Qualitative and quantitative analysis of flavonol compounds and chlorogenic acid in small cranberry fruit samples collected during the ripening period was performed by using UHPLC according to the methodology described by Urbstaite et al. [32]. The analysis of triterpenic compounds was performed according to the technique described by Sedbare et al. [52].

### 3.5. Data Analysis

Statistical analyses were performed using SPSS Statistics 21 (IBM, Chicago, IL, USA) and Microsoft Excel 2016 (Microsoft, Redmond, WA, USA) software. The sample analyses were performed in triplicate. All results are expressed as mean ± standard deviation. A single-factor analysis of variance (ANOVA) along with a post hoc Tukey test was employed for statistical analysis. Differences at *p* < 0.05 were considered to be significant. The coefficient of variation was calculated to determine the variation in the quantitative composition of the studied bioactive compounds between samples of *V. oxycoccos* fruit collected during their ripening period. The correlation was determined by Pearson’s statistical analysis. Correlation coefficients were as follows: 0.00–0.10 was regarded as a negligible correlation, 0.10–0.39 was regarded as a weak correlation, 0.40–0.69 was regarded as a moderate correlation, 0.70–0.89 was regarded as a strong correlation, and 0.90–1.00 was regarded as a very strong correlation [61].

## 4. Conclusions

The study provides new information on the qualitative and quantitative composition and accumulation patterns of biologically active compounds in *V. oxycoccos* fruits growing in natural wetland-type habitats in Lithuanian climate conditions.

The highest total amounts of identified flavonols were determined at the beginning of fruit ripening on September 10. Among individual flavonol compounds, myricetin-3-galactoside and hyperoside predominated. The highest amounts of myricetin-3-galactoside were detected in *V. oxycoccos* fruit samples collected on September 5 and September 18. The highest hyperoside content was found in small cranberry fruit samples collected on September 10.

Meanwhile, the highest total amounts of proanthocyanidins, anthocyanins, and triterpenes were detected in fruit samples collected in the middle of the ripening period (September 16–18). Among anthocyanin group compounds, peonidin-3-O-galactoside and cyanidin-3-O-galactoside predominated. The highest amounts of these compounds were determined in *V. oxycoccos* fruit samples collected on September 18. Among triterpene group compounds, ursolic acid clearly predominated. The highest amount of this acid was determined in small cranberry fruit samples collected on September 16. The in vitro antioxidant activity was found to be strongest in extracts of cranberry fruit collected on September 10.

Studies on the qualitative and quantitative composition of biologically active compounds in cranberry fruit are important for the chemotyping of cranberries growing in natural cenopopulations in Lithuania. Detailed studies on the phytochemical composition of cranberry fruits ensure the preparation of high-quality cranberry raw material, protect cranberry plantations, and allow for a rational use of plant resources. Phytochemical studies on cranberry fruit are important in order to determine the optimum timing for harvesting cranberries and to provide research-based recommendations for the rational harvesting of cranberry fruit in natural habitats.

## Data Availability

All data generated during this study are included in this article.

## Supplementary Materials

The following supporting information can be downloaded at https://www.mdpi.com/article/10.3390/ijms26146956/s1.
